# Supplementary figures and images for: LncRNA-SNPs in a Brazilian Breast Cancer Cohort: A Case-Control Study
Source: Genes (Basel). 2023 Apr 25;14(5):971. doi: 10.3390/genes14050971 (PMC10217596; doi:10.3390/genes14050971)

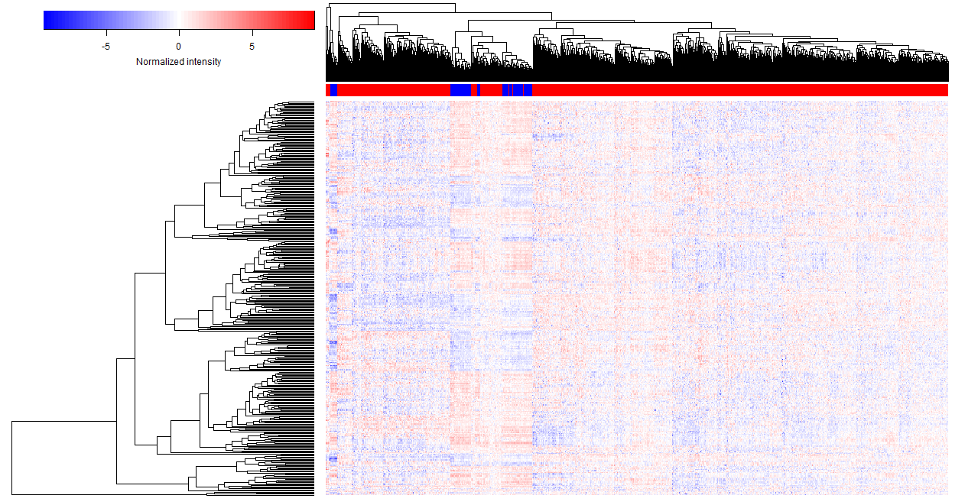

Supplement: Supplementary file 1 [file genes-14-00971-s001.zip › Sup Figure S1.png]
